# High susceptibility to varicella among urban and rural pregnant women in South India: a brief report

**DOI:** 10.1017/S0950268821000492

**Published:** 2021-02-26

**Authors:** Leeberk Raja Inbaraj, Sindhulina Chandrasingh, Nalini Arun Kumar, Jothi Suchitra, Abi Manesh

**Affiliations:** 1 Division of Community Health and Family Medicine, Bangalore Baptist Hospital, Bangalore, Karnataka 560024, India; 2 Department of Microbiology, Bangalore Baptist Hospital, Bangalore, Karnataka 560024, India; 3 Department of Obstetrics and Gynaecology, Bangalore Baptist Hospital, Bangalore, Karnataka 560024, India; 4 Department of Obstetrics and Gynecology, Rural Development Trust Hospital, Bathalapalli, Ananantapur, Andhrapradesh, India; 5 Department of Infectious Diseases, Christian Medical College, Vellore, India

**Keywords:** PPV, pregnancy, self-reported varicella, varicella susceptibility

## Abstract

Varicella infection during pregnancy has serious and/or difficult implications and in some cases lethal outcome. Though epidemiological studies in developing countries reveal that a significant proportion of patients may remain susceptible during pregnancy, such an estimate of susceptible women is not known in India. We designed this study to study the prevalence and factors associated with susceptibility to varicella among rural and urban pregnant women in South India. We prospectively recruited 430 pregnant women and analysed their serum varicella IgG antibodies as surrogates for protection. We estimated seroprevalence, the validity of self-reported history of chickenpox and factors associated with varicella susceptibility. We found 23 (95% CI 19.1–27.3) of women were susceptible. Nearly a quarter (22.2%) of the susceptible women had a history of exposure to chickenpox anytime in the past or during the current pregnancy. Self-reported history of varicella had a positive predictive value of 82.4%. Negative history of chickenpox (adjusted prevalence ratio (PR) 1.85, 95% CI 1.15–3.0) and receiving antenatal care from a rural secondary hospital (adjusted PR 4.08, 95% CI 2.1–7.65) were significantly associated with susceptibility. We conclude that high varicella susceptibility rates during pregnancy were noted and self-reported history of varicella may not be a reliable surrogate for protection.

## Introduction

Varicella infection during pregnancy has serious and/or difficult implications and in some cases lethal outcome. Varicella-associated pneumonia and death are more common among pregnant women [1]. Foetal varicella infection carries up to a 2% risk of congenital varicella syndrome (CVS) characterised by limb, eye and brain abnormalities [2]. Varicella behaves as a childhood disease in temperate regions while it affects significant numbers of adolescents and adults in tropical regions. Large-scale seroepidemiological studies evaluating varicella susceptibility in India are lacking. In a study evaluating 778 health science students with a median age of 21 years, 25.8% were susceptible [3]. In another study involving 5000 nurses with age between 21 and 30 years, 28% were susceptible [4]. An epidemic investigation in South India revealed that 24% of the people infected were 16 years and older. Similarly, 63% of the people infected in rural North India were at least 15 years with a mean age of 23.4 years [5, 6]. Data from epidemics in various parts of India also highlight the high attack rate among age groups 16–25 years suggesting susceptibility [5–7]. Hence, a high proportion of women of childbearing age likely remain susceptible in India. However, this proportion has not been studied so far.

Varicella in pregnancy poses some unique challenges. Avoiding exposure to varicella is difficult as the infectivity period starts a few days before the onset of the characteristic rash. Second, varicella immunoglobulin (VZIg), the treatment of choice, is not widely available in India and most developing countries. Finally, risk assessment for foetal anomalies involves the studies of the foetal blood or amniotic fluid and advanced sonological studies, which require considerable expertise and infrastructure.

An effective, safe, live attenuated Oka strain varicella vaccine has been available globally since 1995[8]. Before the vaccine was widely available in the private sector, 3.4% and 31.1% among the urban and rural Indian population remained susceptible, suggesting significant epidemiological differences within the country [9]. Another study across four cities of India reported 31.8% of the population were susceptible to the age-related increase in seroprevalence rate from 1 to 40 years [10]. Epidemiological studies in developing countries reveal that a significant proportion (Sri Lanka – 34%, Iran – 10. 7%, Egypt – 11.7%) of patients may remain susceptible during pregnancy [11–13]. As for our knowledge, varicella susceptibility rate and positive predictive value (PPV) of the history of chickenpox among pregnant women have not been evaluated in the Indian subcontinent. We attempted to address those questions in our study. Currently, India does not have a vaccine policy with regard to varicella-zoster virus (VZV) and vaccination against varicella is not included in the National Immunisation Program and findings from our study could provide newer insights.

## Materials and methods

### Study setting and participants

We conducted a cross-sectional study of pregnant women attending antenatal clinics in Bangalore Baptist Hospital, a tertiary care centre in Bangalore, Karnataka and Rural Development Trust Hospital, a rural secondary hospital in Bathelapalli, Andhra Pradesh between October and December 2019. The former conducts approximately 3000 deliveries with 7000 pregnant women registered for antenatal care in a year while the latter conducts approximately 4200 deliveries with 9000 registered pregnant women in a year. Considering the seroprevalence of 66% in Sri Lanka, the sample size was calculated as 430 (d – 5%, design effect – 1.2) [4]. Pregnant women with a confirmed pregnancy by either a positive urine pregnancy test or ultrasound with a period of amenorrhea for more than 4 weeks were recruited after informed consent.

### Data collection and sample processing

Data were collected using a pre-tested interviewer-administered questionnaire during their routine visit to the hospital for antenatal care. A positive history of varicella was defined as a report of a generalised blistering rash with fever at least 2 weeks before sample collection. Positive vaccination history was defined as recalling having at least one dose of varicella-containing vaccine at least 2 weeks before sample collection.

The presence of anti-varicella IgG in serum samples was determined by the quantitative anti-VZV IgG ELISA (enzyme linked immunosorbent assay) from Euroimmun (Lübeck, Germany) using highly purified VZV antigens of strain Ellen coated on the microtiter wells. The optical density measurement and calculations were done on an automated ELISA equipment as per the manufacturer's instructions. As per the manufacturer's recommendation, the results were interpreted as positive when  ≥ 110 IU/l and negative when <80 IU/l. Results between 80 and 110 IU/l were considered equivocal. Ten positive and 10 negative samples were tested in parallel in a reference laboratory for verification of accuracy.

For added quality assurance of the ELISA runs, 10% random sample repeat of the entire specimen collection was performed within runs. Samples that initially tested equivocal were re-tested. The samples which remained equivocal again were considered susceptible for statistical analysis.

### Statistical analysis

Data were entered into Microsoft Excel 2010 version and analysed using SPSS version 20.0 (Chicago, Illinois, USA). The terms ‘susceptibility’ and ‘immunity’ are used here to represent the absence or presence of varicella-specific IgG, respectively. Seroprevalence was calculated as the number of cases positive for varicella IgG divided by the number of examined sera. Socio-demographic factors were tested for their association with susceptibility using *χ*^2^ test. Based on the bivariate analysis, we assumed that a variable with *P* < 10% was a confounder. A binary logistic regression was done to adjust for potential confounders such as age (≤30/>30 years), place of residence (rural/urban), education (lower/higher), husband's education (lower/higher), history of chickenpox (yes/no), centre for receiving antenatal care (urban tertiary/rural secondary) with susceptibility and reported as adjusted prevalence ratios with 95% confidence intervals. General assumptions of logistic regression were considered and we did not assume any interactions or collinearity. *P* < 0.05 was considered significant. Goodness of fit was assessed using Hosmer–Lemeshow statistic, Cox and Snell *R*^2^, and Nagelkerke *R*^2^, which are described at the bottom of Table 2. The presence of varicella IgG was considered as gold standard and sensitivity, specificity, negative and positive predictive values were calculated for a positive history of infection and reported with 95% confidence intervals.

## Results

We prospectively recruited 430 pregnant women from 18 to 39 (25.3 ± 4.3) years of age. Almost half of them (54.7%) resided in rural areas, and 56.7% were primigravida. Half of the women (49.8%) had completed higher education (above high school), and 84.9% were housewives. More than half (51.6%) gave a positive history of previous chickenpox, and 8.1% were uncertain about chickenpox infection in the past (Table 1).

**Table 1.** Factors associated with VZV serological status

Factors	Categories	Serological status		Total	*P* value
		Positive	Negative/equivocal		
Age in years	≤30	281(75.1)	93 (24.9)	374	0.01*
	>30	50 (89.3)	6 (10.7)	56	
Location	Rural	170 (72.3)	65 (27.7)	235	0.01*
	Urban	161 (82.6)	34 (17.4)	195	
Centre of receiving ANC	RDT (rural)	140 (65.7)	73 (34.3)	213	0.00*
	BBH (urban)	191 (88)	26 (12)	217	
Gravida	Primi	190 (77.9)	54 (22.1)	244	0.61
	Multi	141 (75.8)	45 (24.2)	186	
No. of living children	≤1	315 (77.8)	90 (22.2)	405	0.11
	>1	16 (64)	9 (36)	25	
Education	Lower	91 (69.5)	40 (30.5)	131	0.01*
	Higher	240 (80.3)	59 (19.7)	299	
Occupation	House wife	276 (75.6)	89 (24.4)	365	0.11
	Working woman	55 (84.6)	10 (15.4)	65	
Husband's education	Lower	88 (70.4)	37 (29.6)	125	0.03*
	Higher	243 (79.7)	62 (20.3)	305	
History of chickenpox	Yes	183 (82.4)	39 (17.6)	222	0.005*
	No/unsure	148 (71.2)	60 (28.8)	208	
History of vaccination	Yes	24 (82.8)	5 (17.2)	29	0.44
	No/unsure	307 (76.6)	94 (23.4)	401	

*Significant *P* value.

VZV IgG was present in 331 (76.9%, 95% CI 72.7–80.9) participants and three (0.6%) had equivocal results. History of chickenpox in the past had 55.3% (95% CI 49.9–60.6) sensitivity, 60.6% (95% CI 50.9–70.2) specificity, PPV of 82.4% (95% CI 77.4–87.4). Of the 222 women with a positive history of varicella, 39 (17.5%) were VZV IgG-negative. A small proportion (6.7%) of the women reported having taken the varicella vaccine in the past.

**Fig. 1.**
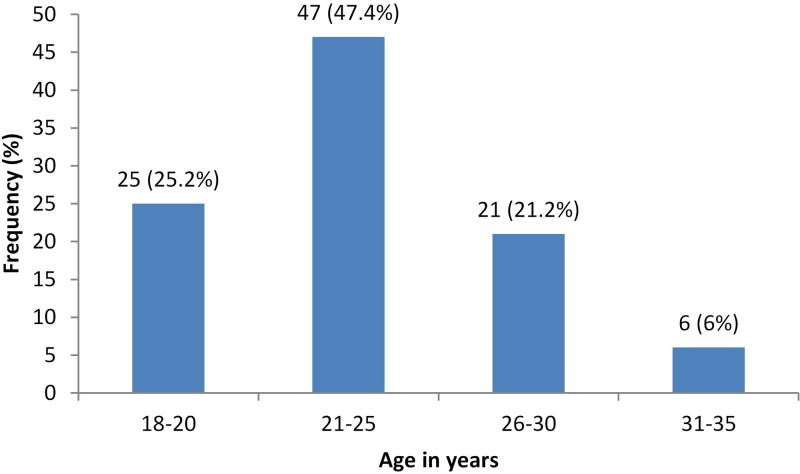
Age specific susceptibility rates for varicella in our study population.

Susceptibility was 1.85-fold (adjusted prevalence ratio −1.85, 95% CI 1.15–3.0, *P* = 0.01) greater with a negative history of chickenpox. Similarly, antenatal care from rural secondary hospital was associated with four times increased rate of susceptibility (adjusted prevalence ratio 4.08, 95% CI 2.1–7.65, *P* = 0.00) compared to those who received antenatal care from a tertiary care urban hospital (Table 2). We also found that the proportion of women with susceptibility was highest in the age group of 21–25 years (47.4%), decreasing with age (Fig. 1). Among those who are susceptible, one-fifth of them (20.2%) gave a history of exposure to varicella sometime in the past and 2.02% of them during the current pregnancy. Only a quarter (24.4%) of the study population reported that they have heard of vaccines for varicella.

**Table 2.** Adjusted prevalence ratio for the susceptibility of varicella infection among pregnant women

Risk factors	Adjusted prevalence ratio	95% CI	*P* value
Age (≤30 years)	0.59	0.33–1.07	0.08
Location (rural)	0.93	0.54–1.61	0.81
Education (lower)	0.86	0.46–1.62	0.65
Husband's education (lower)	0.85	0.45–1.58	0.61
History of chicken pox (no)	1.85	1.15–3.0	0.01*
Centre of receiving care (rural secondary)	4.08	2.1–7.65	0.00*

*R*^2^ = 0.09 (Cox and Snell), 0.31 (Nagelkerke), Model *χ*^2^(6) = 40.73.

*Significant *P* value.

## Discussion

Our study highlights high VZV susceptibility, especially among younger, rural pregnant women in southern India. It also mirrors data from others regarding the high proportion of the susceptible population in India and other South Asian countries [11–14]. This epidemiological pattern is also reflected by the high burden of varicella-zoster among young adults during epidemics [5, 6, 15].

Ongoing varicella transmission, through early adulthood, ensures near-complete protection by around 35 years, as reflected in our study. Unfortunately, this age group is interspersed by periods of pregnancy. The mean age of mothers at the time of first childbirth is around 22 years in most countries of South Asia, and according to the National Family Health Survey-4, 27% of women aged 20–24 married before the age of 18 in India [16, 17]. A significant number of susceptible participants (22.2%) in our study reported varicella exposures during the current pregnancy or in the past.

The poor PPV of self-reported history is an important finding from our study. Typically, the PPV is more than 95% among pregnant women across the studies done in the temperate regions [11, 18]. Many authors have concluded that maternal history can be used safely to exclude patients from serological testing [19, 20]. This is not only true in temperate regions with low susceptibility rates but also in countries like Sri Lanka [11]. This poor PPV (82.4%) noted in our study has important implications in vaccination strategies. Serological testing may be required to identify women who may benefit from the vaccine. While patient factors such as education may play a role, the relative prevalence of diseases that can mimic varicella is also significant. Viral exanthems such as measles and rubella remain prevalent in the Indian setting [21]. India has also emerged as a hotspot of Rickettsial illnesses such as a spotted fever which can be mistaken for varicella [22].

In the context of India and other developing countries, VZIg is not routinely available. While human intravenous immunoglobulin can be used as a substitute, it is expensive, requires multiple doses and often its varicella IgG titres are not known. Ruling out foetal infection after exposure is also difficult in developing countries as the facilities for cordocentesis or amniocentesis for varicella DNA or antibodies and imaging expertise for diagnosing CVS are frequently not available.

Varicella vaccine can overcome these challenges, and has been shown to decrease CVS burden [23]. The cost-benefit of varicella vaccination against VZIg is established in the context of pregnancy [24]. However, being a live attenuated vaccine, it cannot be used during pregnancy and should be administered in the susceptible population in the preconception period. Similarly, the knowledge and uptake of varicella vaccine were also poor among the study population. It implies the need for community sensitisation and periconceptional counselling on varicella vaccines.

To our knowledge, this is the first study to document the susceptibility of varicella among pregnant women in the Indian setting. While the inclusion of participants from an urban and rural setting with an adequate sample size was the strength of our study, it has some limitations. Our study could potentially underestimate varicella exposure during pregnancy, as it shows a point estimate. A follow-up through the period of pregnancy will estimate the risk better. A repeat serology at the end of pregnancy in the susceptible population and documenting foetal outcomes would also be useful. Detailed data on the type of exposure and outcomes following exposure were also not collected.

## Conclusion

Many Indian young pregnant women remain susceptible to VZV. Our data show that self-reported maternal history of varicella cannot be relied on as a surrogate for protection in this high-risk group of patients. We suggest that serologic testing is essential to recognise patients who would benefit from the vaccine in the pre-pregnancy period. We hope these preliminary data, in addition to others, will enable the adoption of the varicella vaccine in the National Immunisation Schedule to protect this high-risk group of patients.

## Data

The datasets used and/or analysed during the current study are available from the corresponding author on reasonable request.
